# A clustering linear combination method for multiple phenotype association studies based on GWAS summary statistics

**DOI:** 10.1038/s41598-023-30415-3

**Published:** 2023-02-28

**Authors:** Meida Wang, Xuewei Cao, Shuanglin Zhang, Qiuying Sha

**Affiliations:** Mathematical Sciences, Michigan Technological University, Houghton, MI USA

**Keywords:** Genetic association study, Genome-wide association studies

## Abstract

There is strong evidence showing that joint analysis of multiple phenotypes in genome-wide association studies (GWAS) can increase statistical power when detecting the association between genetic variants and human complex diseases. We previously developed the Clustering Linear Combination (CLC) method and a computationally efficient CLC (ceCLC) method to test the association between multiple phenotypes and a genetic variant, which perform very well. However, both of these methods require individual-level genotypes and phenotypes that are often not easily accessible. In this research, we develop a novel method called sCLC for association studies of multiple phenotypes and a genetic variant based on GWAS summary statistics. We use the LD score regression to estimate the correlation matrix among phenotypes. The test statistic of sCLC is constructed by GWAS summary statistics and has an approximate Cauchy distribution. We perform a variety of simulation studies and compare sCLC with other commonly used methods for multiple phenotype association studies using GWAS summary statistics. Simulation results show that sCLC can control Type I error rates well and has the highest power in most scenarios. Moreover, we apply the newly developed method to the UK Biobank GWAS summary statistics from the XIII category with 70 related musculoskeletal system and connective tissue phenotypes. The results demonstrate that sCLC detects the most number of significant SNPs, and most of these identified SNPs can be matched to genes that have been reported in the GWAS catalog to be associated with those phenotypes. Furthermore, sCLC also identifies some novel signals that were missed by standard GWAS, which provide new insight into the potential genetic factors of the musculoskeletal system and connective tissue phenotypes.

## Introduction

Over the last decades, genome-wide association studies (GWAS) have been very successful in detecting genetic variants associated with human complex traits or diseases^1–3^. At the same time, a vast majority of GWAS summary statistics obtained from single-trait tests are publicly available, which contain the estimated marginal effect sizes, the corresponding standard deviations, $Z$ scores or p-values. Normally, raw genotypes and phenotypes are not easy to be accessed as a result of privacy concerns and some logistical considerations, thus motivating an extensive interest in developing statistical methods based on GWAS summary statistics^4–6^. On the other hand, because multiple related phenotypes are often measured as indicators for one specific trait, considering the correlated structure between multiple phenotypes and jointly analyzing these phenotypes may increase statistical power in association studies^7–12^.

Recently, many multiple phenotype association tests based on GWAS summary statistics have been proposed. CPASSOC^13^ contains two separate tests (Hom and Het), where Hom is more powerful when the genetic variant has homogeneous effects on the phenotypes; Het is more powerful when heterogeneous effects are present, whereas Monte-Carlo simulations are needed to calculate the p-value of Het when the number of traits is large, which is computationally intensive. SSU^14,15^ is a test statistic based on the sum of squared $Z$ scores, which follows a mixture of chi-squared distributions under the null hypothesis. PCFisher^16^ has the test statistic that combines all p-values of independent principal components using Fisher’s method, where allocates larger weights to PCs with smaller eigenvalues. The classical Wald test^16^ uses the $Z$ score vector and the inverse matrix of the correlation matrix among phenotypes to construct a quadratic test statistic. The adaptive multi-trait association test (aMAT)^17^ builds a group of multi-phenotype association tests (MATs) that may have good performance in a specific scenario and then integrates the testing results adaptively.

In our previous studies, we developed the Clustering Linear Combination (CLC) method^18^ and a computationally efficient CLC (ceCLC) method^19^ to test the association between multiple phenotypes and a genetic variant based on individual level genotypes and phenotypes. Both of these methods perform very well compared with other multiple phenotypes association tests especially for phenotypes that have natural grouping. In this research, we develop a novel approach called CLC based on GWAS summary statistic (sCLC). In sCLC, we use the LD score regression^20,21^ to estimate the correlation matrix among phenotypes. It has been shown that the LD score regression which has been commonly used in recent years can control the potential confounders such as population stratification, unknown sample overlap, cryptic relatedness, and so forth^20–22^. In our simulation studies, we consider a range of simulation settings and compare sCLC with other five commonly used methods for multiple phenotype association studies using GWAS summary statistics to evaluate the performance of sCLC. The simulation results show that sCLC can control the Type I error rate well and has the highest power in most scenarios. We also apply the sCLC method to UK Biobank GWAS summary statistics for 70 related musculoskeletal system and connective tissue phenotypes in the XIII category of UK Biobank. The results show that sCLC identifies the most number of significant SNPs, and most of these SNPs can be matched to the genes that have been reported in the GWAS catalog to be associated with the phenotypes in the XIII category. Furthermore, sCLC also identifies some novel signals that were missed by standard GWAS. The new identified signals may provide new insight into the potential genetic factors of the musculoskeletal system and connective tissue phenotypes.

## Materials and methods

We consider a GWAS with $M$ SNPs and $K$ correlated phenotypes of interest. Each time, a single SNP $j$ is considered, then we repeat the same procedure for all SNPs, $j=1, \cdots ,M$. For SNP $j$, we assume that we have $Z$ score vector ${{\varvec{Z}}}_{{\varvec{j}}}={({Z}_{1j}, {Z}_{2j},\cdots , {Z}_{Kj})}^{T}$ across $K$ phenotypes from GWAS summary statistics. If $Z$ score is not provided, we can compute the $Z$ score as ${Z}_{kj}=\frac{{\widehat{\beta }}_{kj}}{\widehat{se}({\widehat{\beta }}_{kj})}$, $k=1, \cdots ,K$, where ${\widehat{\beta }}_{kj}$ is the estimated effect size of SNP $j$ on phenotype $k$, and $\widehat{se}({\widehat{\beta }}_{kj})$ is the standard deviation of ${\widehat{\beta }}_{kj}$. Based on the GWAS summary statistics, we propose the following sCLC method.

Firstly, sCLC uses the LD score regression (LDSC)^20,21^ to estimate the correlation matrix among phenotypes, denoted by ${\varvec{R}}$. Specifically, consider the pair of phenotypes $s$ and $k$, the bivariate LDSC^20^ regresses the pairwise product of $Z$ scores on the LD scores, the expected value of ${Z}_{sj}{Z}_{kj}$ is:

$$E\left( {Z_{sj} Z_{kj} } \right) = G_{g} l_{j} + \rho_{sk} ,$$

where ${G}_{g}$ is related to the genetic covariance between phenotypes $s$ and $k$; ${l}_{j}$ is the LD score of SNP $j$ which can be obtained from the reference panel^20,21^; and ${\rho }_{sk}$ is the correlation between phenotypes $s$ and $k$. Therefore, the bivariate LDSC^20^ can be applied to each pair of phenotypes, and the estimated intercepts ${\rho }_{sk}$ are used to estimate the off-diagonal elements of ${\varvec{R}}$. When $s=k$, it reduces to the univariate LDSC^21^ for each phenotype and the estimated intercepts are used to estimate the diagonal elements of ${\varvec{R}}$. In this procedure, all $M$ SNPs are used to estimate ${\varvec{R}}$, and the LD scores for SNPs can be obtained from the reference panel, such as the 1000 Genome Project^23^. Moreover, LDSC can control potential confounders such as population stratification, unknown sample overlap, cryptic relatedness, and so forth^20–22^.

Secondly, similar to CLC^18^, we use the hierarchical clustering approach with similarity matrix $${\varvec{R}}$$ and dissimilarity matrix $$1-{\varvec{R}}$$ to partition the original $$K$$ phenotypes into $$L$$ disjoint clusters ($$L=\mathrm{1,2},\dots ,K)$$. The agglomerative hierarchical clustering starts with each phenotype as a singleton cluster ($$L=K)$$ and then successively merges pairs of clusters that have the smallest distance (highest similarity) until all clusters have been merged into a single cluster that contains all phenotypes ($$L=1)$$^24^. Because we consider a single SNP $$j$$ and multiple phenotypes at a time, the notation $${{\varvec{Z}}}_{{\varvec{j}}}$$ can be simplified by $${\varvec{Z}}$$. After applying the hierarchical clustering method to partition the original $$K$$ phenotypes into $$L$$ disjoint clusters ($$L=\mathrm{1,2},\dots ,K)$$, we define a $$K\times L$$ matrix $${\varvec{B}}$$ with the $${(k,l)}^{th}$$ element equals 1 if the $$k$$th phenotype belongs to the $$l$$th cluster, otherwise it equals 0. Then the CLC test statistic to test the association between the $$K$$ phenotypes and a SNP with $$L$$ clusters is given by:

$${T}_{CLC}^{L}={\left({\varvec{W}}{\varvec{Z}}\right)}^{T}{\left({\varvec{W}}{\varvec{R}}{{\varvec{W}}}^{T}\right)}^{-1}\left({\varvec{W}}{\varvec{Z}}\right),$$

where ${\varvec{W}}={{\varvec{B}}}^{T}{{\varvec{R}}}^{-1}$. ${T}_{CLC}^{L}$ follows a ${\chi }^{2}$ distribution with degrees of freedom $L$ under the null hypothesis. We denote the p-value of ${T}_{CLC}^{L}$ by ${p}_{L}$ for $1\le L\le K$.

Finally, we use Cauchy combination^25,26^ to integrate the p-values obtained from the second step for all possible number of clusters, ${p}_{L}$ for $1\le L\le K$. The test statistic of sCLC for a SNP is defined as the linear combination of the transformed p-values divided by $K$ (all possible number of clusters), which is given by

$${T}_{sCLC}=\frac{1}{K}{\sum }_{L=1}^{K}\mathrm{tan}\left((0.5-{p}_{L})\pi \right).$$

Under the null hypothesis, ${p}_{L}$ follows a standard uniform distribution, so $\mathrm{tan}\left((0.5-{p}_{L})\pi \right)$ has a standard Cauchy distribution. Because ${p}_{1}, \cdots , {p}_{K}$ correspond to each possible number of clusters for $K$ phenotypes, there exists a correlated structure between them. Liu et al.^25,26^ showed that a weighted sum of “correlated” standard Cauchy variables still has an approximately Cauchy tail, and the influence of the correlated structure on the tail is quite limited because of the heaviness of the Cauchy tail. Therefore, ${T}_{sCLC}$ is approximately standard Cauchy distributed. Based on the cumulative density distribution of the standard Cauchy distribution, the p-value of ${T}_{sCLC}$ can be approximated by $0.5-\left(\mathrm{arctan}\left({T}_{sCLC}\right)/\pi \right)$.

## Comparison of methods

To better demonstrate the performance of the sCLC approach, we compare sCLC with other five methods for multiple phenotype association studies using GWAS summary statistics: SSU^14,15^, Hom^13^, PCFisher^16^, Wald^16^, and aMAT^17^. Below, we briefly summarize these five methods, where ${\varvec{Z}}$ score vector and the phenotypic correlation matrix ${\varvec{R}}$ are the same as we define previously.

### SSU

The test statistic of SSU is ${T}_{SSU}={{\varvec{Z}}}^{T}{\varvec{Z}}$ and the distribution of ${T}_{SSU}$ can be well approximated by $a{\chi }_{d}^{2}+b$ with $a=\frac{\sum_{i=1}^{K}{c}_{i}^{3}}{\sum_{i=1}^{K}{c}_{i}^{2}}$, $b=\sum_{i=1}^{K}{c}_{i}-\frac{{(\sum_{i=1}^{K}{c}_{i}^{2})}^{2}}{\sum_{i=1}^{K}{c}_{i}^{3}}$, and $d=\frac{{(\sum_{i=1}^{K}{c}_{i}^{2})}^{3}}{{(\sum_{i=1}^{K}{c}_{i}^{3})}^{2}}$, where ${c}_{i}$ s are the eigenvalues of ${\varvec{R}}$. The p value of ${T}_{SSU}$ can be obtained by $p({\chi }_{d}^{2}>({T}_{SSU}-b)/a)$. Note that the degrees of freedom of ${T}_{SSU}$ may be less than $K$ with highly correlated phenotypes.

### Hom

Assume that there are summary statistics of GWASs from $J$ cohorts with $K$ traits. Let ${T}_{ijk}$ be a summary statistic for the $i$th SNP, $j$th cohort, and $k$th trait. Let ${{\varvec{T}}}_{i}=(T_{i11}, \ldots,T_{iJ1}, \ldots,T_{i1K}, \ldots,T_{iJK})^T$. For simplification, we omit the SNP index, then ${\varvec{T}}=(T_{11}, \ldots,T_{J1}, \ldots,T_{1K}, \ldots,T_{JK})^T$ represents a vector of test statistics for single SNP-trait association tests. The test statistic of Hom is ${S}_{Hom}=\frac{{{\varvec{e}}}^{{\varvec{T}}}{({\varvec{R}}{\varvec{V}})}^{-1}{\varvec{T}}{({{\varvec{e}}}^{{\varvec{T}}}{({\varvec{R}}{\varvec{V}})}^{-1}{\varvec{T}})}^{T}}{{{\varvec{e}}}^{{\varvec{T}}}{({\varvec{V}}{\varvec{R}}{\varvec{V}})}^{-1}{\varvec{e}}}$, which follows a ${\chi }^{2}$ distribution with one degree of freedom, where ${{\varvec{e}}}^{{\varvec{T}}}=(1, \ldots,1)$ is a vector of length $J\times K$ with all elements being 1, ${\varvec{V}}$ is a diagonal matrix of weights ${w}_{jk}=\sqrt{{n}_{j}}$, and ${n}_{j}$ is the sample size in the $j$th cohort. In this study, we consider $J=1$ cohort to compare Hom with other methods.

### PCFisher

Assume that the spectral decomposition of ${\varvec{R}}$ is ${\varvec{R}}={\sum }_{m=1}^{K}{\lambda }_{m}{\mathbf{u}}_{m}{\mathbf{u}}_{m}^{T}$, where ${\lambda }_{1}\ge {\lambda }_{2}\ge \cdots \ge {\lambda }_{K}>0$ are the eigenvalues of ${\varvec{R}}$, and ${\mathbf{u}}_{m}$ is the eigenvector corresponding to the $m$ th largest eigenvalue ${\lambda }_{m}$. We assume that the $K$-dimensional vector of the summary statistics ${\varvec{Z}}\sim N({\varvec{\mu}}, {\varvec{R}})$. It can be shown that^16^ ${PC}_{m}={\mathbf{u}}_{m}^{T}{\varvec{Z}}\sim N\left({\mathbf{u}}_{m}^{T}{\varvec{\mu}}, {\lambda }_{m}\right), 1\le m\le K.$ The non-centrality parameter (*ncp*) of ${\mathrm{PC}}_{m}$ under the alternative hypothesis is $nc{p}_{m}={({\mathbf{u}}_{m}^{T}{\varvec{\mu}})}^{2}/{\lambda }_{m}$. PCFisher^16^ combines p-values of all $K$ independent principal components using Fisher’s method with its null distribution and the test statistic is given by $\mathrm{PCFihser}=-2\sum_{m=1}^{K}\mathrm{log}({p}_{m})\sim {\chi }_{2K}^{2}$.

### Wald

The test statistic of Wald test is defined as ${T}_{Wald}={{\varvec{Z}}}^{T}{{\varvec{R}}}^{-1}{\varvec{Z}}$. Assume that the spectral decomposition of ${\varvec{R}}$ is ${\varvec{R}}={\varvec{U}}{\varvec{\Lambda}}{{\varvec{U}}}^{T}={\sum }_{m=1}^{K}{\lambda }_{m}{\mathbf{u}}_{m}{\mathbf{u}}_{m}^{T}$, then the test statistic can be written as ${T}_{Wald}={{\varvec{Z}}}^{T}{{\varvec{R}}}^{-1}{\varvec{Z}}={\left({{\varvec{U}}}^{T}{\varvec{Z}}\right)}^{T}{{\varvec{\Lambda}}}^{-1}\left({{\varvec{U}}}^{T}{\varvec{Z}}\right)={\sum }_{m=1}^{K}\frac{{PC}_{m}^{2}}{{\lambda }_{m}}\sim {\chi }_{K}^{2}$. So, the Wald test is a special quadratic PC-based test^16^.

### aMAT

The method was developed to deal with potential (near) singularity problem of ${\varvec{R}}$. The singular value decomposition (SVD) of ${\varvec{R}}$ is ${\varvec{R}}={\varvec{U}}{\varvec{\Sigma}}{{\varvec{U}}}^{T}$. A modified pseudoinverse ${{\varvec{R}}}_{\gamma }^{+}$ is calculated by ${{\varvec{R}}}_{\gamma }^{+}={\varvec{U}}{{\varvec{\Sigma}}}_{\gamma }^{+}{{\varvec{U}}}^{T}$, where ${\Sigma }_{\gamma }^{+}$ is formed from $\Sigma$ by taking the reciprocal of the largest $m$ singular values $\sigma_{1} , \ldots ,\sigma_{m}$, and setting all other elements to zero, where $m$ is the largest integer that satisfies ${\sigma }_{1}/{\sigma }_{m}<\gamma$. The test statistic of ${\mathrm{MAT}}_{(\gamma )}$ is defined as ${T}_{{\mathrm{MAT}}_{(\gamma )}}={{\varvec{Z}}}^{T}{{\varvec{R}}}_{\gamma }^{+}{\varvec{Z}}$. Because the optimal value of $\gamma$ is unknown, aMAT combines the results from a class of MAT tests, ${T}_{\mathrm{aMAT}}=\underset{\gamma \in\Gamma }{\mathrm{min}}{p}_{\mathrm{MAT}(\upgamma )}$, where ${p}_{\mathrm{MAT}(\upgamma )}$ is the p value of ${\mathrm{MAT}}_{(\gamma )}$, and $\Gamma =\left(1, 10, 30, 50\right)$. Finally, a Gaussian copula approximation is applied to calculate the p-value of aMAT. Therefore, aMAT is analogous to a PC-based method which restricts the analysis to the top $m$ axes of the largest variation^17^.

## Results

### Simulation design

Based on a widely used simulation procedure^17,27^, we generate $Z$ scores from a multivariate normal distribution $N({\varvec{\mu}}, {\varvec{R}})$. We consider two different correlation matrix structures: (1) ${\varvec{R}}$ is the sample correlation matrix of 70 related musculoskeletal system and connective tissue phenotypes in the UK Biobank (details of the 70 phenotypes are described in the Application to UK Biobank summary statistics); and (2) ${\varvec{R}}$ is generated based on the Autoregressive model (AR(1) model)^28^ for 40 phenotypes, where ${\varvec{R}}=Bdiag({{\varvec{R}}}_{1}, {{\varvec{R}}}_{2}, {{\varvec{R}}}_{3}, {{\varvec{R}}}_{4})$, a block diagonal matrix, with ${{\varvec{R}}}_{1}={{\varvec{R}}}_{3}=\left({r}_{sk}\right)={\rho }^{|s-k|}$ and ${{\varvec{R}}}_{2}={{\varvec{R}}}_{4}=-{\rho }^{|s-k|}$. We use $\rho =0.1$ in the simulation studies.

To investigate how the estimation error of ${\varvec{R}}$ may affect on the testing results, similar to Wu^17^, we consider two cases in the 70 phenotypic correlation matrix structure. In the first case, we suppose that ${\varvec{R}}$ is known and perform our proposed method, sCLC, and all competing methods based on ${\varvec{R}}$. In the second case, we suppose that ${\varvec{R}}$ is unknown and the estimated phenotypic correlation matrix is approximated by ${\varvec{R}}$ with a small white noise $N(0, \delta )$, denoted by ${\varvec{R}}\left(\delta \right).$ We choose $\delta ={10}^{-5}$ and ${\delta =10}^{-4}$ in the simulation studies, and use ${\varvec{R}}(\delta )$ in the association tests for all the methods.

To evaluate Type I error rate of sCLC, we generate ${10}^{8}$ ${\varvec{Z}}$ score vectors under the null hypothesis (${\varvec{\mu}}=0$) and choose different significant levels. In order to evaluate power, we generate ${10}^{4}$ ${\varvec{Z}}$ score vectors under an alternative with different effect size vector ${\varvec{\mu}}$ in four scenarios. In the first two scenarios, we assume that the SNP impacts on phenotypes with the same direction. Scenario 3 considers different directions of effects on phenotypes. Scenario 4 is a sparse simulation model, where a SNP impacts on a small proportion of phenotypes. The significant level of $5\times {10}^{-8}$ is chosen for the power evaluation.

Scenario 1: Generate $${\varvec{\mu}}=\beta(1/K, 2/K, \ldots,1)^T$$.

Scenario 2: Generate $${\varvec{\mu}}= ( {\underbrace {{0,0,...,0}}_{{K/2}},\underbrace {{\beta ,\beta ,...,\beta }}_{{K/2}}})^{T}$$.

Scenario 3: Generate ${\varvec{\mu}}= (\beta _{{11}} , \ldots ,\beta _{{1k}} ,\beta _{{21}} , \ldots ,\beta _{{2k}} ,\beta _{{31}} , \ldots ,\beta _{{3k}} ,\beta _{{41}} , \ldots ,\beta _{{4k}} ,\beta _{{51}} , \ldots ,\beta _{{5k}} )^{T}$, where ${\beta }_{11}=\cdots ={\beta }_{1k}= {\beta }_{21}=\cdots ={\beta }_{2k}=0, {\beta }_{31}=\cdots ={\beta }_{3k}={\beta }_{41}=\cdots ={\beta }_{4k}=\beta$, ${(\beta }_{51},\cdots ,{\beta }_{5k})=-\frac{2\beta }{k+1}(1, \cdots , k)$, and $k=K/5.$

Scenario 4: Generate ${\varvec{\mu}}= (\beta _{{11}} , \ldots, \beta _{{1k}} ,\beta _{{21}} , \ldots ,\beta _{{2k}} ,\beta _{{31}} , \ldots ,\beta _{{3k}} , \ldots, \beta _{{14,1}} , \ldots ,\beta _{{14,k}} )^{T} .$ ${\beta }_{11}=\cdots ={\beta }_{1k}= {\beta }_{21}=\cdots ={\beta }_{2k}=\cdots ={\beta }_{\mathrm{13,1}}=\cdots ={\beta }_{13,k}=0,$ ${(\beta }_{\mathrm{14,1}},\cdots ,{\beta }_{14, k})=\frac{2\beta }{k+1}(1, \cdots , k)$, and $k=K/14.$

### Simulation results

#### Type I error rates

Table 1 shows the estimated Type I error rates at different significance levels for all six methods with the phenotypic correlation matrix ${\varvec{R}}$ of 70 phenotypes. The Type I error rates with the correlation matrix ${\varvec{R}}({10}^{-5})$ and ${\varvec{R}}({10}^{-4})$ of 70 phenotypes are recorded in Tables S1 and S2. From these tables, we can see that the sCLC approach can control the Type I error rates very well at different significant levels $\alpha$, which indicates that it is a valid test. Among the five competing methods, SSU yields inflated Type I error rates when $\alpha$ is smaller and the other four methods can control Type I error rates very well. Table S3 shows the estimated Type I error rates at different significance levels for all six methods with the phenotypic correlation structure for the 40 phenotypes. We observe that all methods can well-control Type I error rates.

**Table 1:** The estimated Type I error rates at different significance levels for the six methods with the phenotypic correlation structure for the 70 phenotypes.

${\alpha }$	$\boldsymbol{{1\times 10}^{-3}}$	$\boldsymbol{1\times {10}^{-4}}$	$\boldsymbol{1\times {10}^{-5}}$	$\boldsymbol{1\times {10}^{-6}}$	$\boldsymbol{1\times {10}^{-7}}$
SSU	$1.05\times {10}^{-3}$	$\boldsymbol{1.13\times {10}^{-4}}$	$\boldsymbol{1.25\times {10}^{-5}}$	$\boldsymbol{1.61\times {10}^{-6}}$	$\boldsymbol{2.29\times {10}^{-7}}$
sCLC	$1.07\times {10}^{-3}$	$1.05\times {10}^{-4}$	$1.06\times {10}^{-5}$	$1.17\times {10}^{-6}$	$7.98\times {10}^{-8}$
Hom	$1.00\times {10}^{-3}$	$9.82\times {10}^{-5}$	$1.01\times {10}^{-5}$	$9.47\times {10}^{-7}$	$9.97\times {10}^{-8}$
Wald	$1.01\times {10}^{-3}$	$1.00\times {10}^{-4}$	$9.98\times {10}^{-6}$	$1.17\times {10}^{-6}$	$1.7\times {10}^{-7}$
aMAT	$9.97\times {10}^{-4}$	$1.00\times {10}^{-4}$	$1.02\times {10}^{-5}$	$1.17\times {10}^{-6}$	$1.3\times {10}^{-7}$
PCFisher	$1.00\times {10}^{-3}$	$9.90\times {10}^{-5}$	$1.01\times {10}^{-5}$	$1.09\times {10}^{-6}$	$1.5\times {10}^{-7}$

The bold-faced values indicate that the type I error rates cannot be controlled.

#### Power comparisons

**Figure 1:**
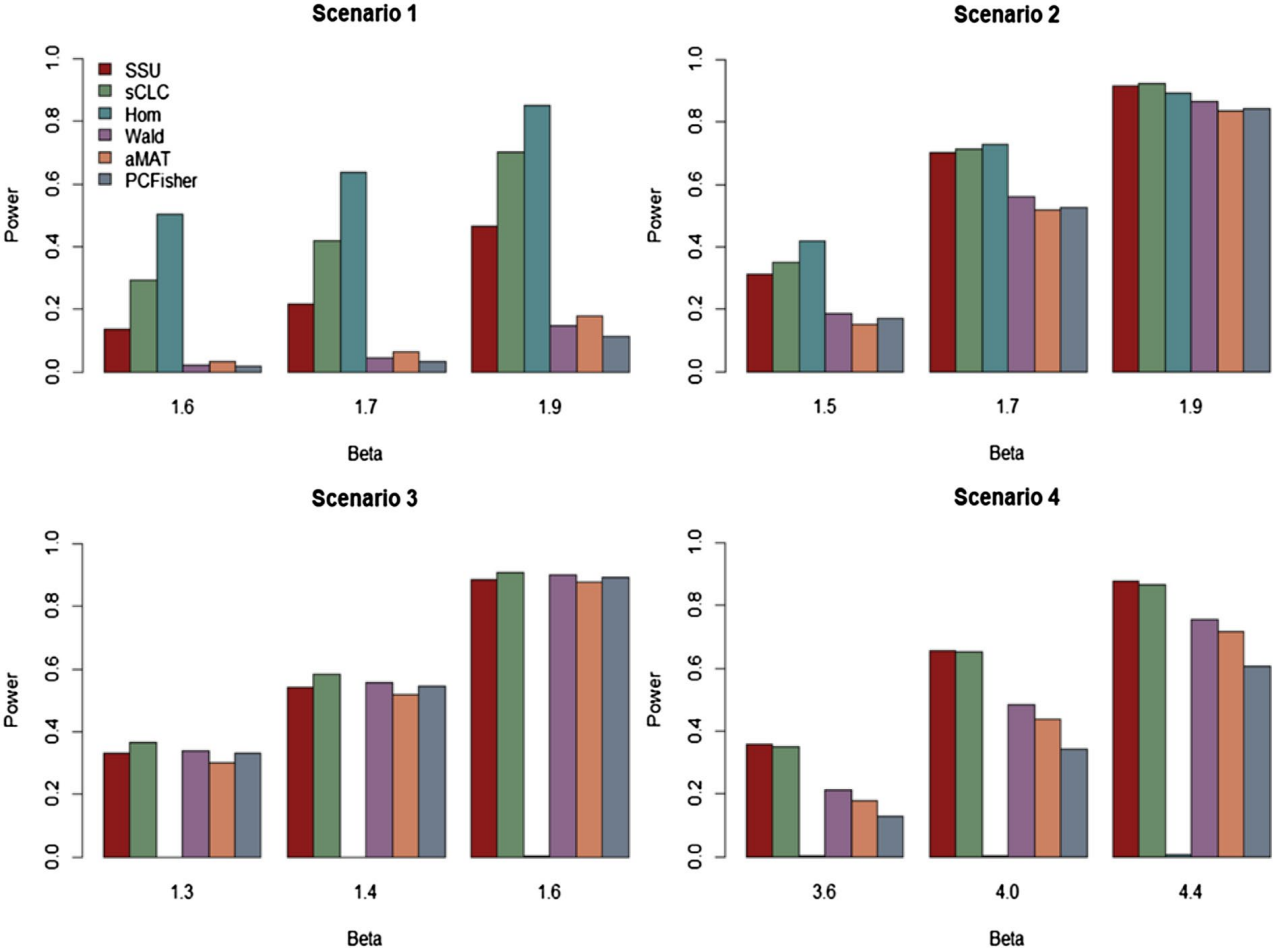
Power comparisons of the six methods, SSU, sCLC, Hom, Wald, aMAT, and PCFisher for the phenotypic correlation structure of the 70 phenotypes at a significant level of $5\times {10}^{-8}.$

Power comparison results of the six methods under four scenarios with the phenotypic correlation matrix ${\varvec{R}}$ of 70 phenotypes are presented in Fig. 1. Figures S1 and S2 show the power comparisons of the six methods with the correlation matrix ${\varvec{R}}({10}^{-5})$ and ${\varvec{R}}({10}^{-4})$ of 70 phenotypes, respectively. From these figures, we can observe that (1) when SNPs have homogeneous effects on the phenotypes (scenarios 1 and 2), our proposed method sCLC, as well as Hom and SSU have higher power than the other three PC-based methods (Wald, aMAT, and PCFisher); whereas all the methods have comparable powers except for Hom when the SNP affects on phenotypes in different directions. (2) The power of Hom dramatically reduces and almost is zero in scenarios 3, while sCLC and SSU are robust to the direction of the genetic effect on the phenotypes. (3) sCLC and SSU are more powerful than other methods when a SNP affects on a small proportion of phenotypes (scenario 4), and Hom is less powerful in this case. (4) In all of the four scenarios, the power patterns observed in Figs. S1 and S2 are very close to that of Fig. 1, indicating that the estimation errors (noise $\delta$) of ${\varvec{R}}$ have little influence on the powers for all the methods. Figure S3 shows the power comparisons of the six methods with the phenotypic correlation structure for the 40 phenotypes. sCLC is still more powerful than the other five methods under all four scenarios.

## Application to UK biobank summary statistics

Connective tissue dysplasia (CTD) and musculoskeletal disorders^29–31^, such as Systemic Lupus Erythematosus (SLE), Sjögren Syndrome (SS), and Rheumatoid Arthritis (RA), may influence the physical activity or movement of patients. These kinds of diseases seriously affect the quality of life of people and have been reported to be potentially affected by genetic factors^32^. In this paper, we consider the GWAS summary statistics in the XIII category of UK Biobank with 70 musculoskeletal system and connective tissue phenotypes to detect potential genetic factors.

The UK Biobank is a large long-term biobank study which has recruited almost half a million participants in the UK, enrolled at ages from 40 to 69^33^. Sequenced genotypes for 488,377 participants with 784,256 variants in autosomal chromosomes were extracted by UK Biobank dataset^34^. Similar to Liang et al.^28^, we first perform quality controls (QCs) on genotypes and individuals by using PLINK 1.9^35^. We remove SNPs with missing rates larger than 5%, p-values from Hardy–Weinberg equilibrium exact test less than ${10}^{-6}$, and minor allele frequency (MAF) less than 5%. In addition, we screen out individuals with missing genotype rate larger than 5% and without sex information. After these pre-processing, there are 466,580 individuals with 288,647 genetic variants left.

On the other hand, the phenotypes that coded by International Classification of Diseases, the 10th Revision (ICD-10) codes are considered in our study. We truncate the full ICD-10 code to the UK Biobank ICD-10 level 3 code (http://biobank.ndph.ox.ac.uk/showcase/field.cgi?id=41202) to define Electronic Health Record (EHR)-derived phenotypes. When the individual has the truncated ICD-10 code recorded for a specific phenotype, the corresponding EHR-derived phenotype for that individual will be coded as 1, otherwise it will be 0 (1 for cases and 0 for controls). In the XIII category, we only consider phenotypes with more than 200 cases and there are a total of 72 unique phenotypes, such as rheumatoid arthritis (M06.9) and Systemic Lupus Erythematosus (M32.9). Table S4 lists the ICD-10 code, the name of the disease, heritability, and case–control ratio for each of the 72 phenotypes. Since our proposed method is a population-based method and cannot be applied to a mixed population due to population stratification, we analyze 409,672 individuals with the white British ancestry. Similar to Liang et al.^28^, we also exclude individuals who are marked as outliers for heterozygosity, and have been identified to have more than ten third-degree relatives or closer, etc. The final dataset includes $N=\mathrm{322,607}$ individuals with $M=\mathrm{288,647}$ common variants across $K=72$ phenotypes for analyses. All the phenotypes are adjusted by 13 covariates, including age, sex, genotyping array, and the first 10 genetic principal components (PCs).

To apply our method, we first calculate the GWAS summary statistics for the 72 phenotypes based on $\mathrm{288,647}$ SNPs. We observed that all of the 72 phenotypes have extremely unbalanced case–control ratios, where the largest case–control ratio is 0.03937 for Gonarthrosis (M17.9) and the smallest case–control ratio is 0.000658 for Lumbar and other intervertebral disk disorders with myelopathy (M51.0). Therefore, we use the saddlepoint approximation (SPA)^36^ to calculate the adjusted $Z$ scores. For the $j$th SNP and $k$th phenotype $\left( {j = 1, \ldots ,M, k = 1, \ldots ,K} \right)$, we calculate the score test statistic^37^ ${S}_{kj}={\sum }_{i=1}^{N}({Y}_{ik}-{\overline{Y} }_{k}){G}_{ij}$, where ${\overline{Y} }_{k}={\sum }_{i=1}^{n}{Y}_{ik}/N$. ${Y}_{ik}$ denotes the $k$th phenotype for the $i$th individual, ${G}_{ij}$ denotes the $j$th SNP for the $i$th individual ($i = 1, \ldots ,N$). The adjusted $Z$-score is defined as ${Z}_{kj}=sign({S}_{kj})\sqrt{{F}_{Chi}^{-1}(1-{p}_{kj})}$, where ${F}_{Chi}( )$ denotes the cumulative density function of ${\chi }_{1}^{2}$ and ${p}_{kj}$ is the p-value of ${S}_{kj}$ obtained using SPA^36^. Based on the adjusted $Z$-scores, we then apply LDSC to estimate the correlation matrix among phenotypes. We run the single-trait LDSC^21^ to estimate the diagonal elements for each phenotype, and the off-diagonal elements are estimated by the cross-trait LDSC^20^. Two phenotypes M79.6 (Enthesopathy of lower limb) and M67.8 (Other specified disorders of synovium and tendon) are excluded in this procedure because the estimators of their heritability are out of bounds. Therefore, there are a total of 70 phenotypes in the simulation studies and real data analysis. The phenotypic correlation matrix only needs to be estimated once for all SNPs. Finally, we apply our proposed sCLC method and the other five methods to test the association between each of 288,647 SNPs and 70 phenotypes, and the commonly used genome-wide significant level $\alpha =5\times {10}^{-8}$ is considered.

**Figure 2:**
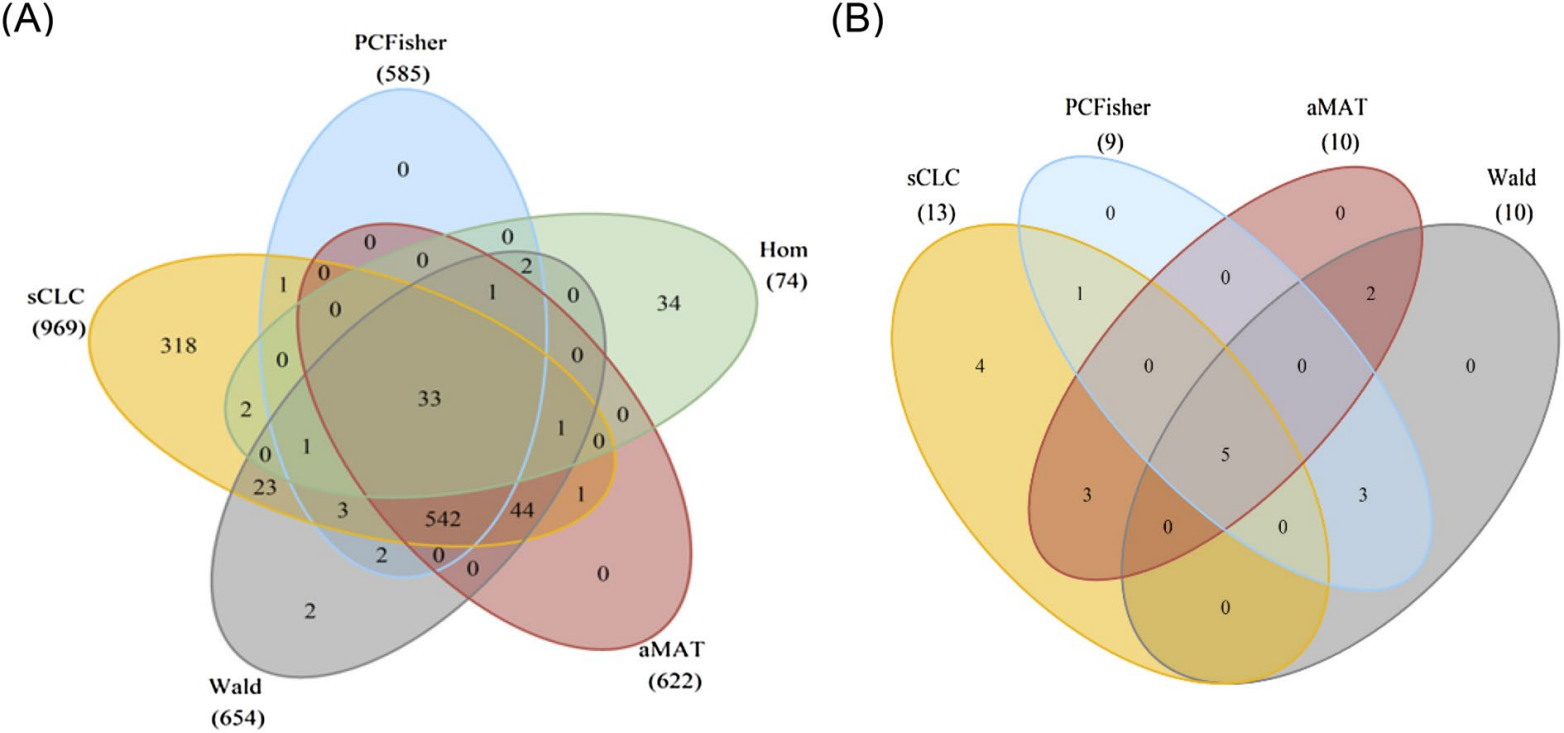
Venn diagram. (**A**) The number of significant SNPs identified by the five methods. (**B**) The number of lead SNPs identified by sCLC, Wald, aMAT, and PCFisher.

**Figure 3:**
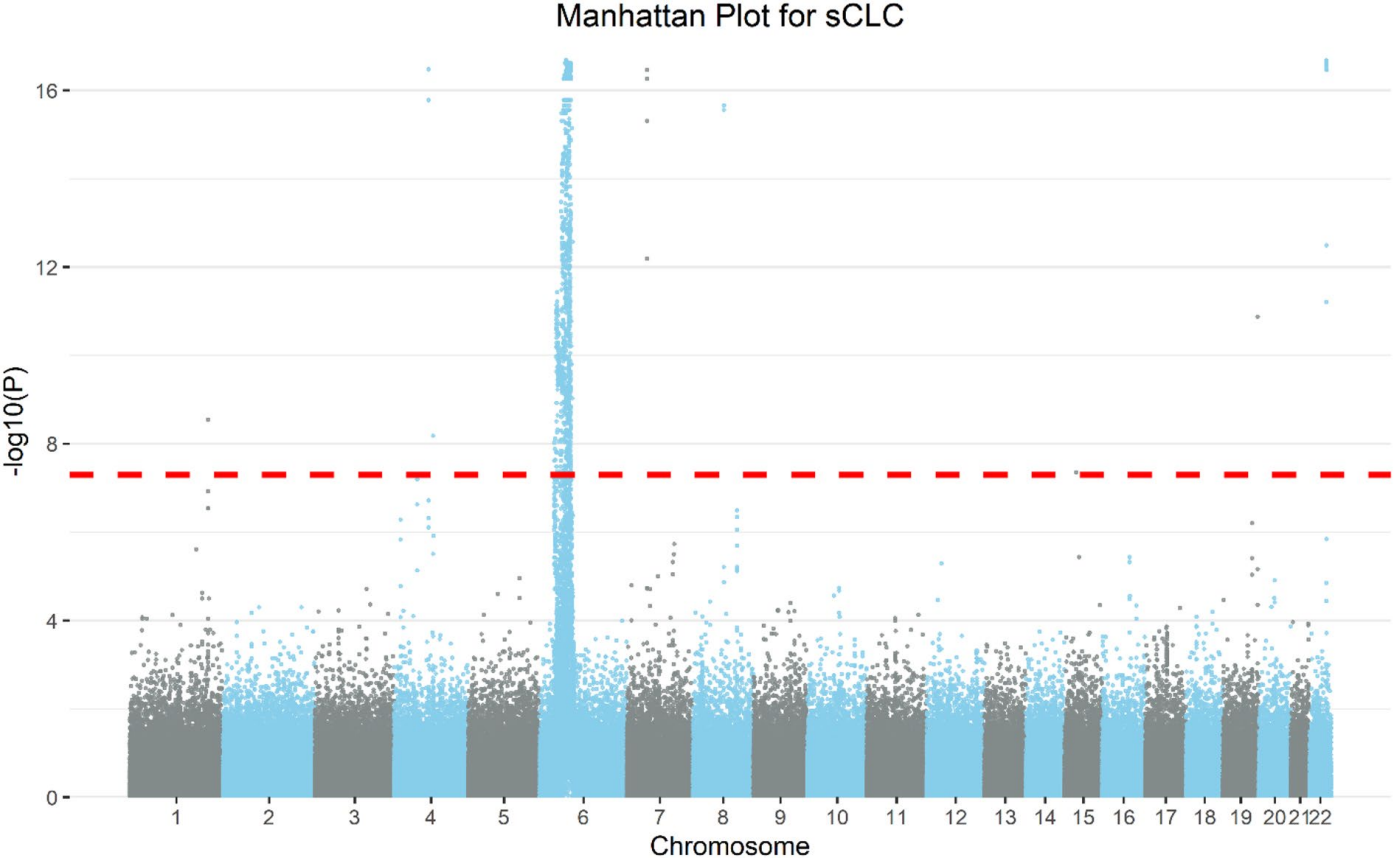
Manhattan Plot from the results of sCLC using multiple phenotypes based on the phenotypes on the UK Biobank XIII category. Each SNP ordered by the genomic position is represented in the x-axis and the association strength with the transformed p-values $-{\mathrm{log}}_{10}\left(p\right)$ is represented in the y-axis.

Among all the six methods, sCLC identifies the largest number of SNPs (969), where Hom identifies 74 SNPs, SSU identifies 872 SNPs, Wald test identifies 654 SNPs, aMAT identifies 622 SNPs, and PCFisher identifies 585 SNPs. Figure 2A shows the Venn Diagram for five methods except for SSU, since SSU cannot control Type I error rates in our simulation studies. There are 33 SNPs identified by all five methods, and 318 SNPs only identified by sCLC. Figure 3 shows the Manhattan plot from the sCLC test results, in which 947 out of 969 SNPs are located in chromosome 6. To evaluate the 969 SNPs identified by sCLC, we first map those SNPs to genes, and we use the commonly used UCSC reference gene file (https://hgdownload-test.gi.ucsc.edu/goldenPath/hg19/bigZips/genes/). Each gene has a position interval. A SNP can be mapped to a gene if its position is within the interval or 20 kb downstream or 20 kb upstream from the interval. These 969 SNPs can be mapped to 235 genes. From the results, we find that 746 out of 969 SNPs can be matched to the genes that have been reported to be associated with the Chapter XIII phenotypes in GWAS catalog. Moreover, among 318 SNPs only identified by sCLC, 229 SNPs can be mapped to the genes that have been reported to be associated with those phenotypes.

However, SNPs within the same LD block are highly correlated and are more likely to be mapped to the same gene. For example, 205 out of 969 identified SNPs are mapped to gene TSBP1-AS1, which is associated with 10 phenotypes in the XIII category; other genes such as NOTCH4, HLA-DRA, and HLA-DRB1 also have many identified SNPs mapped on them. Hence, we are also interested in the independent lead SNPs associated with those phenotypes. We use the Functional Mapping and Annotation (FUMA)^38^ platform to obtain independent lead SNPs and distinct risk loci. Here, the independent lead SNPs are defined as ${r}^{2}<0.1$ and distinct loci are $>$ 250 kb apart. The 969 SNPs identified by sCLC are represented by 13 lead SNPs located in 8 distinct risk loci; the 654 SNPs identified by Wald are represented by 10 lead SNPs located in 6 distinct risk loci; the 622 SNPs identified by aMAT are represented by 10 lead SNPs located in 7 distinct risk loci; and the 585 SNPs identified by PCFisher are represented by 10 lead SNPs located in 6 distinct risk loci. Since the MHC region is excluded by FUMA^38^, Hom has no lead SNPs. Figure 2B shows the Venn Diagram of the lead SNPs for sCLC, Wald, aMAT and PCFisher. There are 5 lead SNPs identified by all four methods, and 4 lead SNPs only identified by sCLC. Table 2 shows the details of the summary statistics for all of the 18 independent lead SNPs identified by those four methods. The graying out rows indicate that the SNPs/matched genes have been reported in the GWAS catalog. There are 5 out 13 lead SNPs for sCLC that have not been reported in the GWAS catalog, which may provide us a new insight into the potential genetic factors of the musculoskeletal system and connective tissue phenotypes. Among those 5 SNPs, SNP rs13107325 has the Annotation-Dependent Depletion (CADD) score^39^ greater than 20, which means having a high observed probability of a deleterious variant effect. In addition, we compare the p-values of the 13 independent lead SNPs obtained by sCLC with the minimum p-value (MinP) among 70 p-values for testing the association between a SNP and each of the 70 phenotypes. Table S5 shows the comparison results. There are 6 out of 13 SNPs (graying out) with $\mathrm{MinP}>{5\times 10}^{-8}$, indicating that these six SNPs have no association with any of the 70 phenotypes by univariate association tests. However, by jointly analyzing the 70 phenotypes, sCLC identified these six SNPs indicating that these 6 SNPs have pleiotropic effects on the phenotypes.

**Table 2:** Summary statistics of the independent lead SNPs identified by sCLC, Wald, aMAT, PCFisher.

Chr	SNP	BP	A1	A2	sCLC P	Wald P	aMAT P	PCFisher P	Mapped gene	Reported trait
**1**	**rs4846567**	**219,750,717**	**G**	**T**	**2.88E−09**	**–**	**–**	**–**	**ZC3H11B**	**M19.9; M85.8**
**4**	**rs4148157**	**89,020,934**	**A**	**G**	**1.67E−16**	**–**	**6.54E−14**	**–**	**ABCG2**	**M10.9**
**4**	**rs2231142**	**89,052,323**	**G**	**T**	**–**	**5.16E−17**	**–**	**3.96E−16**	**ABCG2**	**M10.9**
**4**	**rs13107325**	**103,188,709**	**C**	**T**	**6.70E−09**	**–**	**7.46E−09**	**–**	**SLC39A8**	**M19.9**
6	rs13212534	25,983,010	A	G	9.47E−09	–	–	–	TRIM38	
6	rs13195040	27,413,924	A	G	–	9.00E−09	1.80E−08	–	ZNF184	
**6**	**rs13207082**	**27,251,379**	**A**	**G**	**1.08E−10**	**–**	**–**	**2.31E−08**	**POM121L2**	**M85.8**
6	rs67340775	28,304,384	A	G	3.78E−12	–	–	–	ZKSCAN3	
**6**	**rs3117425**	**29,260,431**	**C**	**T**	**–**	**1.46E−08**	**2.92E−08**	**–**	**OR14J1**	**M72.9**
**6**	**rs404240**	**29,523,957**	**A**	**G**	**1.91E−11**	**–**	**–**	**–**	**GABBR1**	**M32.9; M85.8**
**7**	**rs2598104**	**37,977,249**	**C**	**T**	**5.00E−16**	**1.07E−13**	**2.14E−13**	**5.81E−14**	**EPDR1**	**M72.0; M85.8**
**7**	**rs2290221**	**37,987,632**	**A**	**G**	**–**	**5.32E−20**	**–**	**4.69E−19**	**EPDR1**	**M72.0; M85.8**
7	rs118028828	38,026,155	C	T	5.55E−17	–	2.22E−16	–		
8	rs655028	70,049,047	A	G	2.22E−16	7.08E−16	1.44E−15	4.31E−15		
**19**	**rs34945782**	**57,678,336**	**C**	**T**	**1.34E−11**	**2.16E−08**	**4.32E−08**	**2.42E−08**	**DUXA**	**M72.0; M85.9**
**22**	**rs62228062**	**46,381,234**	**A**	**G**	**–**	**1.74E−35**	**–**	**2.88E−32**	**WNT7B**	**M85.9**
22	rs28698504	46,403,715	A	G	6.23E−12	1.24E−09	2.48E−09	2.06E−08		
**22**	**rs9627391**	**46,447,097**	**C**	**T**	**3.27E−13**	**2.50E−12**	**4.99E−12**	**1.50E−11**	**LINC00899**	**M72.0**

The bold out rows indicate that the SNPs/mapped genes have been reported in the GWAS Catalog. “–” represents that the SNP is not an independent lead SNP for the corresponding method.

**Figure 4:**
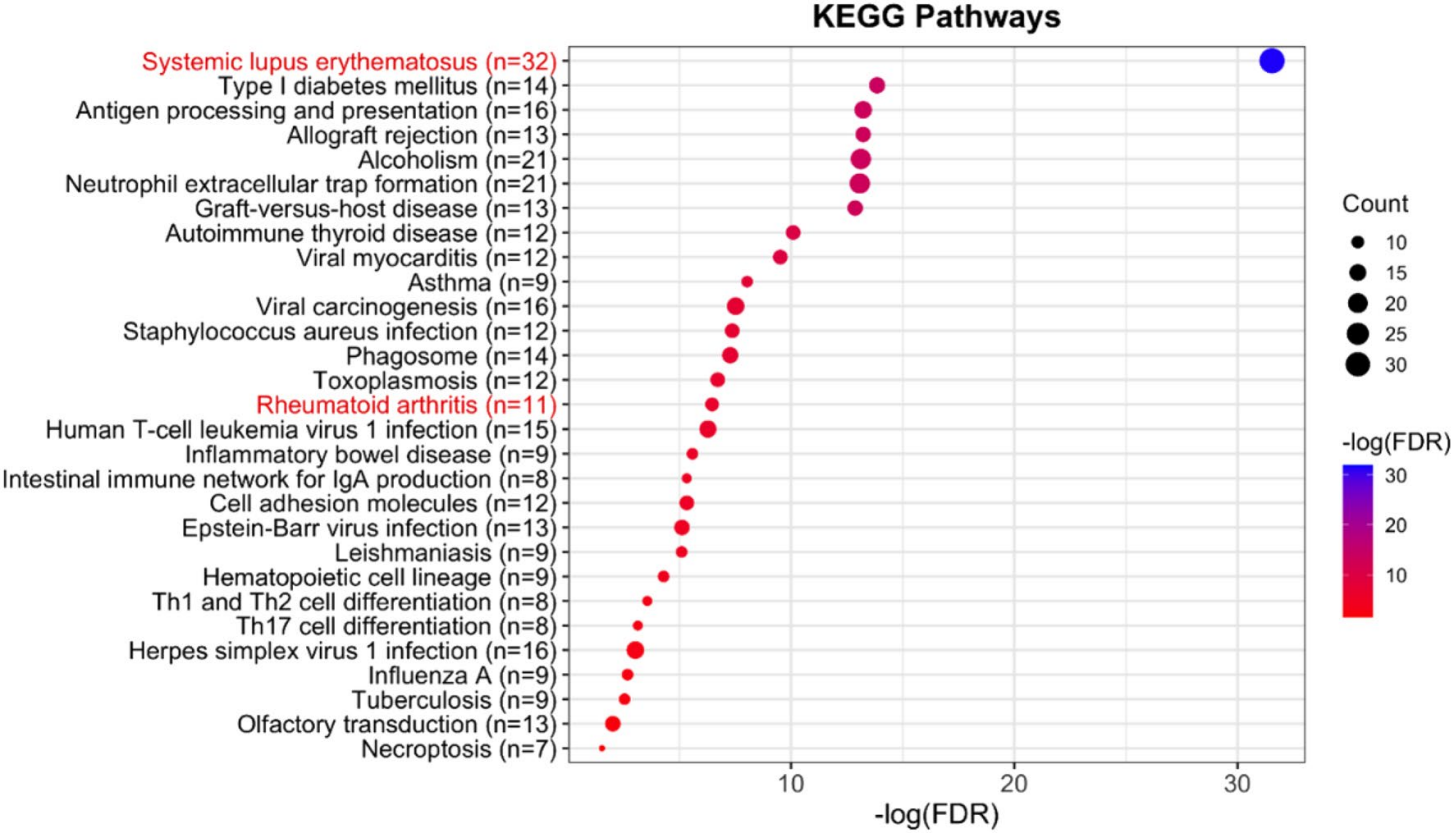
The KEGG pathway enrichment analysis is based on the genes identified by sCLC and the KEGG database. The pathways in red denote the pathways that are related to the diseases of the musculoskeletal system and connective tissue.

In order to better understand the biological meaning behind 235 mapped genes identified by sCLC, similar to Cao et al.^40^, we use DAVID functional annotation software for the Kyoto Encyclopedia of Genes and Genomes (KEGG) pathway enrichment analysis^41,42^. There are 29 significantly enriched pathways identified by sCLC with FDR < 0.05 and enriched gene count > 2 (Fig. 4). From Fig. 4, we can observe that two related pathways significantly enriched, systemic lupus erythematosus (hsa05322; $FDR=2.9\times {10}^{-32}$) and rheumatoid arthritis (hsa05323; $FDR=3.7\times {10}^{-7}$). Especially, there are 32 genes enriched in the systemic lupus erythematosus pathway, including eight genes in HLA-family (*HLA-DMA, HLA-DMB, HLA-DOB, HLA-DQA2, HLA-DQA1, HLA-DRA, HLA-DRB1, HLA-DQB1*), 20 genes in the four core histones (H2A(6): *H2AC6, H2AC13, H2AC14, H2AC15, H2AC16, H2AC17*; H2B(6): *H2BC3, H2BC4, H2BC13, H2BC14, H2BC15, H2BC17*; H3(4): *H3C3, H3C10, H3C11, H3C12*; H4(4): *H4C3, H4C11, H4C12, H4C13*), as well as four genes (*C2, C4B, C4A, TNF*). For the rheumatoid arthritis pathway, sCLC identifies 104 SNPs mapped to 11 genes that are enriched in this pathway, including *HLA-DMA, HLA-DMB, ATP6V1G2, HLA-DRA, LTB, TNF, HLA-DOB, HLA-DQA2, HLA-DRB1, HLA-DQA1*, and *HLA-DQB1*.

## Discussion

In this paper, we propose a multiple-phenotype association test strategy called sCLC which is based on GWAS summary statistics. Through a variety of simulation studies and an application to the UK Biobank XIII category summary statistics, we observed that sCLC is a valid and powerful approach. Specially, sCLC detected some novel signals associated with the musculoskeletal system and connective tissue phenotypes, which provides more evidence to show that those diseases are potentially affected by genetic factors. The sCLC method is also computationally efficient. Since the estimation of the phenotypic correlation matrix ${\varvec{R}}$ is independent of the association test for each SNP, we only need to estimate ${\varvec{R}}$ once by using LDSC for all SNPs. In real data analysis with 288,647 SNPs and 70 phenotypes, after estimation of ${\varvec{R}}$, the running time of sCLC on a computer with 4 Intel Cores @ 3.60 GHz and 16 GB memory is about 4 min 40 s. sCLC as well as many other multiple phenotype association methods, such as the compared methods in this article, test the null hypothesis that a given variant does not contribute to any of the analyzed phenotypes. Therefore, a genetic variant will be identified by these methods even if it is associated with only one phenotype. Hence the identified genetic variants by these methods may not be pleiotropic variants and further analyses are required to interpret the possibility of pleiotropy^43^. This is a limitation of the proposed method in identifying pleiotropic effects. Recently, some methods^43–45^ are proposed to evaluate pleiotropic effects. For example, Schaid et al.^43^ proposed a new statistical method to evaluate pleiotropy using a sequential testing framework. This approach can determine the number of phenotypes associated with a genetic variant and which phenotypes are associated, while accounting for correlations among the phenotypes. SHAHER^44^, a novel framework for analysis of the shared genetic background of correlated phenotypes, can identify genetic factors common for all analyzed phenotypes and specific genetic factors for each phenotype using genetic correlations between phenotypes. PolarMorphism^46^ is a summary-statistic-based framework to map and interpret pleiotropic loci in a joint analysis of multiple phenotypes. It identifies horizontally pleiotropic SNPs by converting the trait-specific SNP effect sizes to polar coordinates.

On the other hand, the hierarchical clustering approach in sCLC is applied to cluster multiple phenotypes based on the phenotypic correlation matrix ${\varvec{R}}$. Therefore, the phenotypes in the same cluster may be affected by non-genetic factors, which may influent the power for disease variant discovery. Instead of using the phenotypic correlation matrix, the genetic correlation matrix among multiple phenotypes^20,21^ can also be used in the hierarchical clustering. Furthermore, considering only the phenotypes with a significant non-zero heritability in the estimation of the genetic correlation matrix may also improve the statistical power in the multiple phenotype association studies. Therefore, we would like to consider using the genetic correlation matrix estimated by the LDSC regression^20^ or using network-based approaches to cluster phenotypes based on shared genetic architectures in our further work^47^.

## Supplementary Information

Supplementary Information.

## Data Availability

UK Biobank data can be accessed by application through http://www.ukbiobank.ac.uk. UK Biobank has approval by the Research Ethics Committee (REC) under approval number 16/NW/0274. UK Biobank obtained participant’s consent for the data to be used for health-related research, and all methods were performed in accordance with the relevant guidelines and regulations.
