# Supplementary material for: A clustering linear combination method for multiple phenotype association studies based on GWAS summary statistics
Source: Sci Rep. 2023 Feb 28;13:3389. doi: 10.1038/s41598-023-30415-3 (PMC9975197; doi:10.1038/s41598-023-30415-3)

**A Clustering Linear Combination Method for Multiple Phenotype Association Studies based on GWAS Summary Statistics**

Meida Wang^1^, Xuewei Cao^1^, Shuanglin Zhang^1^, Qiuying Sha^1*^

^1^ Mathematical Sciences, Michigan Technological University, Houghton, MI, USA.

^*^ Corresponding author

E-mail: qsha@mtu.edu

**Table S1** The estimated Type I error rates for different significance levels of the six methods with the phenotypic correlation structure of the 70 phenotypes for $\delta={10}^{-5}$.

| $\boldsymbol{\alpha}$ | $\boldsymbol{1\times10}^{\boldsymbol{-3}}$ | $\boldsymbol{1\times}\boldsymbol{10}^{\boldsymbol{-4}}$ | $\boldsymbol{1\times}\boldsymbol{10}^{\boldsymbol{-5}}$ | $\boldsymbol{1\times}\boldsymbol{10}^{\boldsymbol{-6}}$ | $\boldsymbol{1\times}\boldsymbol{10}^{\boldsymbol{-7}}$ |
| --- | --- | --- | --- | --- | --- |
| SSU | $1.04\times{10}^{-3}$ | $\boldsymbol{1.12\times}\boldsymbol{10}^{\boldsymbol{-4}}$ | $\boldsymbol{1.26\times}\boldsymbol{10}^{\boldsymbol{-5}}$ | $\boldsymbol{1.43\times}\boldsymbol{10}^{\boldsymbol{-6}}$ | $\boldsymbol{2.29\times}\boldsymbol{10}^{\boldsymbol{-7}}$ |
| sCLC | $1.07\times{10}^{-3}$ | $1.05\times{10}^{-4}$ | $1.06\times{10}^{-5}$ | $1.03\times{10}^{-6}$ | $8.98\times{10}^{-8}$ |
| Hom | $9.97\times{10}^{-4}$ | $1.00\times{10}^{-4}$ | $9.72\times{10}^{-6}$ | $8.81\times{10}^{-7}$ | $8.98\times{10}^{-8}$ |
| Wald | $1.00\times{10}^{-3}$ | $9.90\times{10}^{-5}$ | $1.01\times{10}^{-5}$ | $1.04\times{10}^{-6}$ | $1.01\times{10}^{-7}$ |
| aMAT | $9.96\times{10}^{-4}$ | $1.00\times{10}^{-4}$ | $1.02\times{10}^{-5}$ | $9.87\times{10}^{-7}$ | $1.10\times{10}^{-7}$ |
| PCFisher | $1.00\times{10}^{-3}$ | $1.00\times{10}^{-4}$ | $9.54\times{10}^{-6}$ | $9.87\times{10}^{-7}$ | $9.12\times{10}^{-8}$ |

Notes: the bold-faced values indicate that the type I error rates cannot be controlled.

**Table S2** The estimated Type I error rates for different significance levels of the six methods with the phenotypic correlation structure of the 70 phenotypes for $\delta={10}^{-4}$.

| $\boldsymbol{\alpha}$ | $\boldsymbol{1\times10}^{\boldsymbol{-3}}$ | $\boldsymbol{1\times}\boldsymbol{10}^{\boldsymbol{-4}}$ | $\boldsymbol{1\times}\boldsymbol{10}^{\boldsymbol{-5}}$ | $\boldsymbol{1\times}\boldsymbol{10}^{\boldsymbol{-6}}$ | $\boldsymbol{1\times}\boldsymbol{10}^{\boldsymbol{-7}}$ |
| --- | --- | --- | --- | --- | --- |
| SSU | $1.05\times{10}^{-3}$ | $\boldsymbol{1.13\times}\boldsymbol{10}^{\boldsymbol{-4}}$ | $\boldsymbol{1.22\times}\boldsymbol{10}^{\boldsymbol{-5}}$ | $\boldsymbol{1.42\times}\boldsymbol{10}^{\boldsymbol{-6}}$ | $\boldsymbol{1.59\times}\boldsymbol{10}^{\boldsymbol{-7}}$ |
| sCLC | $1.07\times{10}^{-3}$ | $1.06\times{10}^{-4}$ | $1.02\times{10}^{-5}$ | $8.92\times{10}^{-7}$ | $8.94\times{10}^{-8}$ |
| Hom | $1.00\times{10}^{-3}$ | $9.96\times{10}^{-5}$ | $9.83\times{10}^{-6}$ | $8.92\times{10}^{-7}$ | $9.91\times{10}^{-8}$ |
| Wald | $9.94\times{10}^{-4}$ | $9.91\times{10}^{-5}$ | $1.03\times{10}^{-5}$ | $1.04\times{10}^{-6}$ | $1.09\times{10}^{-7}$ |
| aMAT | $9.90\times{10}^{-4}$ | $9.94\times{10}^{-5}$ | $1.00\times{10}^{-5}$ | $1.04\times{10}^{-6}$ | $9.92\times{10}^{-8}$ |
| PCFisher | $1.00\times{10}^{-3}$ | $1.01\times{10}^{-4}$ | $1.02\times{10}^{-5}$ | $1.14\times{10}^{-6}$ | $8.94\times{10}^{-8}$ |

Notes: the bold-faced values indicate that the type I error rates cannot be controlled.

**Table S3** The estimated Type I error rates for different significance levels of the six methods with the phenotypic correlation structure of the 40 phenotypes.

| $\boldsymbol{\alpha}$ | $\boldsymbol{1\times10}^{\boldsymbol{-3}}$ | $\boldsymbol{1\times}\boldsymbol{10}^{\boldsymbol{-4}}$ | $\boldsymbol{1\times}\boldsymbol{10}^{\boldsymbol{-5}}$ | $\boldsymbol{1\times}\boldsymbol{10}^{\boldsymbol{-6}}$ | $\boldsymbol{1\times}\boldsymbol{10}^{\boldsymbol{-7}}$ |
| --- | --- | --- | --- | --- | --- |
| SSU | $1.01\times{10}^{-3}$ | $1.02\times{10}^{-4}$ | $1.04\times{10}^{-5}$ | $8.95\times{10}^{-7}$ | $8.77\times{10}^{-8}$ |
| sCLC | $1.07\times{10}^{-3}$ | $1.06\times{10}^{-4}$ | $1.05\times{10}^{-5}$ | $9.21\times{10}^{-7}$ | $7.89\times{10}^{-8}$ |
| Hom | $1.01\times{10}^{-3}$ | $1.02\times{10}^{-4}$ | $9.89\times{10}^{-6}$ | $9.30\times{10}^{-7}$ | $8.77\times{10}^{-8}$ |
| Wald | $1.00\times{10}^{-3}$ | $1.01\times{10}^{-4}$ | $9.81\times{10}^{-6}$ | $8.77\times{10}^{-7}$ | $1.05\times{10}^{-7}$ |
| aMAT | $9.89\times{10}^{-4}$ | $1.02\times{10}^{-4}$ | $9.77\times{10}^{-6}$ | $8.33\times{10}^{-7}$ | $7.02\times{10}^{-8}$ |
| PCFisher | $1.00\times{10}^{-3}$ | $1.01\times{10}^{-4}$ | $1.01\times{10}^{-5}$ | $8.87\times{10}^{-7}$ | $6.14\times{10}^{-8}$ |

**Table S4** Short description of the 72 EHR-derived phenotypes after pre-processing.

| **Disease** | **ICD-10 code** | **heritability** | **# of cases** | **# of controls** | **case-control ratio** |
| --- | --- | --- | --- | --- | --- |
| Rheumatoid arthritis, unspecified | M06.9 | 0.0041 | 1605 | 321002 | 0.005 |
| Other psoriatic arthropathies | M07.3 | -0.0015 | 311 | 322296 | 0.000965 |
| Gout, unspecified | M10.9 | 0.0078 | 1225 | 321382 | 0.003812 |
| Polyarthritis, unspecified | M13.0 | 0.0038 | 215 | 322392 | 0.000667 |
| Arthritis, unspecified | M13.9 | 0.0068 | 3164 | 319443 | 0.009905 |
| Primary generalised (osteo)arthrosis | M15.0 | 0.0025 | 380 | 322227 | 0.001179 |
| Polyarthrosis, unspecified | M15.9 | 0.0073 | 2666 | 319941 | 0.008333 |
| Primary coxarthrosis, bilateral | M16.0 | 0.0029 | 611 | 321996 | 0.001898 |
| Other primary coxarthrosis | M16.1 | 0.0071 | 2660 | 319947 | 0.008314 |
| Coxarthrosis, unspecified | M16.9 | 0.0144 | 6497 | 316110 | 0.020553 |
| Primary gonarthrosis, bilateral | M17.0 | 0.0043 | 999 | 321608 | 0.003106 |
| Other primary gonarthrosis | M17.1 | 0.0118 | 3900 | 318707 | 0.012237 |
| Gonarthrosis, unspecified | M17.9 | 0.023 | 12218 | 310389 | 0.039364 |
| Other primary arthrosis of first carpometacarpal joint | M18.1 | -0.0008 | 229 | 322378 | 0.00071 |
| Arthrosis of first carpometacarpal joint, unspecified | M18.9 | 0.002 | 733 | 321874 | 0.002277 |
| Arthrosis, unspecified | M19.9 | 0.0088 | 4241 | 318366 | 0.013321 |
| Hallux valgus (acquired) | M20.1 | 0.0125 | 5108 | 317499 | 0.016088 |
| Hallux rigidus | M20.2 | 0.0086 | 1184 | 321423 | 0.003684 |
| Other hammer toe(s) (acquired) | M20.4 | 0.0088 | 1478 | 321129 | 0.004603 |
| Other deformities of toe(s) (acquired) | M20.5 | 0.0037 | 1365 | 321242 | 0.004249 |
| Acquired deformity of toe(s), unspecified | M20.6 | 0.0024 | 234 | 322373 | 0.000726 |
| Chondromalacia patellae | M22.4 | 0.0012 | 327 | 322280 | 0.001015 |
| Derangement of meniscus due to old tear or injury | M23.2 | 0.0005 | 1265 | 321342 | 0.003937 |
| Other meniscus derangements | M23.3 | 0.0007 | 551 | 322056 | 0.001711 |
| Loose body in knee | M23.4 | -0.0006 | 434 | 322173 | 0.001347 |
| Other internal derangements of knee | M23.8 | -0.0038 | 697 | 321910 | 0.002165 |
| Effusion of joint | M25.4 | -0.0029 | 205 | 322402 | 0.000636 |
| Pain in joint | M25.5 | 0.002 | 1342 | 321265 | 0.004177 |
| Osteophyte | M25.7 | -0.0043 | 342 | 322265 | 0.001061 |
| Other giant cell arteritis | M31.6 | 0.001 | 285 | 322322 | 0.000884 |
| Systemic lupus erythematosus, unspecified | M32.9 | -0.0002 | 237 | 322370 | 0.000735 |
| Sicca syndrome [Sjogren] | M35.0 | 0.0008 | 378 | 322229 | 0.001173 |
| Polymyalgia rheumatica | M35.3 | 0.0066 | 886 | 321721 | 0.002754 |
| Scoliosis, unspecified | M41.9 | 0.0014 | 263 | 322344 | 0.000816 |
| Ankylosing spondylitis | M45 | 0.0046 | 293 | 322314 | 0.000909 |
| Ankylosing spondylitis (Site unspecified) | M45.X9 | 0.0022 | 240 | 322367 | 0.000744 |
| Other spondylosis | M47.8 | 0.0019 | 755 | 321852 | 0.002346 |
| Spondylosis, unspecified | M47.9 | 0.0009 | 688 | 321919 | 0.002137 |
| Spinal stenosis | M48.0 | 0.0014 | 485 | 322122 | 0.001506 |
| Cervical disk disorder with myelopathy | M50.0 | 0.0039 | 305 | 322302 | 0.000946 |
| Cervical disk disorder with radiculopathy | M50.1 | 0.0024 | 386 | 322221 | 0.001198 |
| Other cervical disk displacement | M50.2 | 0.0024 | 263 | 322344 | 0.000816 |
| Other cervical disk degeneration | M50.3 | -0.0023 | 298 | 322309 | 0.000925 |
| Lumbar and other intervertebral disk disorders with myelopathy | M51.0 | 0.0032 | 212 | 322395 | 0.000658 |
| Lumbar and other intervertebral disk disorders with radiculopathy | M51.1 | 0.0036 | 2545 | 320062 | 0.007952 |
| Other specified intervertebral disk displacement | M51.2 | 0.0024 | 2031 | 320576 | 0.006335 |
| Other specified intervertebral disk degeneration | M51.3 | 0.0061 | 1972 | 320635 | 0.00615 |
| Sacrococcygeal disorders, not elsewhere classified | M53.3 | 0.001 | 207 | 322400 | 0.000642 |
| Cervicalgia | M54.2 | 0.0039 | 737 | 321870 | 0.00229 |
| Sciatica | M54.3 | -0.0026 | 686 | 321921 | 0.002131 |
| Lumbago with sciatica | M54.4 | -4.22E-06 | 241 | 322366 | 0.000748 |
| Low back pain | M54.5 | 0.0111 | 2799 | 319808 | 0.008752 |
| Dorsalgia, unspecified | M54.9 | 0.0017 | 1679 | 320928 | 0.005232 |
| Trigger finger | M65.3 | 0.0048 | 1326 | 321281 | 0.004127 |
| Synovitis and tenosynovitis, unspecified | M65.9 | -0.0006 | 292 | 322315 | 0.000906 |
| Ganglion | M67.4 | 0.0031 | 2209 | 320398 | 0.006895 |
| Other specified disorders of synovium and tendon | M67.8 | 0.0001 | 418 | 322189 | 0.001297 |
| Trochanteric bursitis | M70.6 | 0.0026 | 355 | 322252 | 0.001102 |
| Palmar fascial fibromatosis [Dupuytren] | M72.0 | 0.021 | 1873 | 320734 | 0.00584 |
| Adhesive capsulitis of shoulder | M75.0 | 0.0072 | 1306 | 321301 | 0.004065 |
| Rotator cuff syndrome | M75.1 | 0.0108 | 2751 | 319856 | 0.008601 |
| Calcific tendinitis of shoulder | M75.3 | 0.0028 | 255 | 322352 | 0.000791 |
| Impingement syndrome of shoulder | M75.4 | 0.0082 | 3764 | 318843 | 0.011805 |
| Bursitis of shoulder | M75.5 | -0.0016 | 463 | 322144 | 0.001437 |
| Other shoulder lesions | M75.8 | 0.0032 | 1182 | 321425 | 0.003677 |
| Lateral epicondylitis | M77.1 | 0.0013 | 311 | 322296 | 0.000965 |
| Rheumatism, unspecified | M79.0 | 0.002 | 297 | 322310 | 0.000921 |
| Pain in limb | M79.6 | 0.0003 | 1004 | 321603 | 0.003122 |
| Fibromyalgia | M79.7 | 0.0015 | 463 | 322144 | 0.001437 |
| Other specified soft tissue disorders | M79.8 | 0.0051 | 636 | 321971 | 0.001975 |
| Osteoporosis, unspecified | M81.9 | 0.0081 | 2187 | 320420 | 0.006825 |
| Other specified disorders of bone density and structure | M85.8 | -0.0001 | 252 | 322355 | 0.000782 |

**Table S5** The comparison of the p-values for the 13 independent lead SNPs obtained by sCLC with the minimum p-value (MinP) among 70 p-values obtained by testing the association between a SNP and each of 70 phenotypes.

| **Locus** | **SNP** | **CHR** | **BP** | **A1** | **A2** | **sCLC P** | **Reported trait** | **MinP** |
| --- | --- | --- | --- | --- | --- | --- | --- | --- |
| 1 | rs4846567 | 1 | 219750717 | G | T | 2.88E-09 | M19.9; M85.8 | 1.47E-05 |
| 2 | rs4148157 | 4 | 89020934 | A | G | 1.67E-16 | M10.9 | 1.49E-25 |
| 3 | rs13107325 | 4 | 103188709 | C | T | 6.70E-09 | M19.9 | 7.58E-06 |
| 4 | rs13212534 | 6 | 25983010 | A | G | 9.47E-09 |  | 1.02E-06 |
| 4 | rs13207082 | 6 | 27251379 | A | G | 1.08E-10 | M85.8 | 6.00E-06 |
| 4 | rs67340775 | 6 | 28304384 | A | G | 3.78E-12 |  | 6.74E-06 |
| 4 | rs404240 | 6 | 29523957 | A | G | 1.91E-11 | M32.9; M85.8 | 7.95E-06 |
| 5 | rs2598104 | 7 | 37977249 | C | T | 5.00E-16 | M72.0; M85.8 | 2.05E-27 |
| 5 | rs118028828 | 7 | 38026155 | C | T | 5.55E-17 |  | 4.45E-33 |
| 6 | rs655028 | 8 | 70049047 | A | G | 2.22E-16 |  | 6.01E-24 |
| 7 | rs34945782 | 19 | 57678336 | C | T | 1.34E-11 | M72.0; M85.9 | 2.88E-17 |
| 8 | rs28698504 | 22 | 46403715 | A | G | 6.23E-12 |  | 3.14E-19 |
| 8 | rs9627391 | 22 | 46447097 | C | T | 3.27E-13 | M19.9; M85.8 | 2.35E-22 |

Notes: the graying out SNPs indicate that they are identified by sCLC but missed by the univariant association tests.

**Figure S1** Power comparisons of the six methods, SSU, sCLC, Hom, Wald, aMAT, and PCFisher for the phenotypic correlation structure of the 70 phenotypes for $\delta={10}^{-5}$ at a significant level of $5\times{10}^{-8}.$


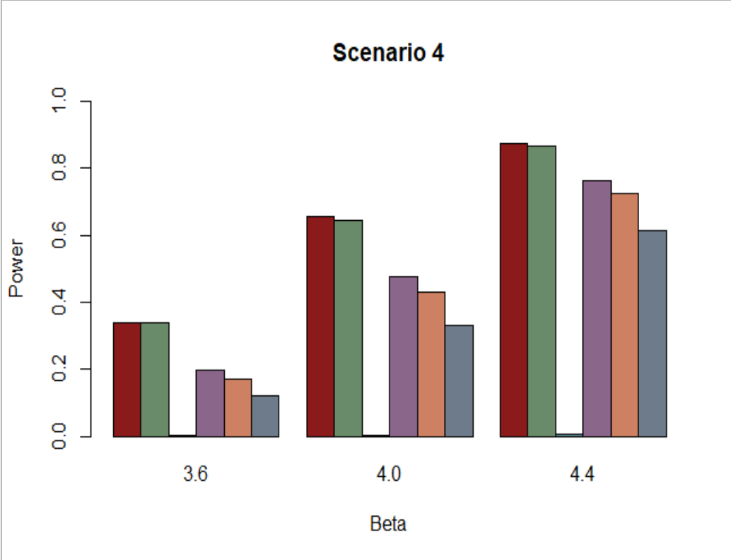

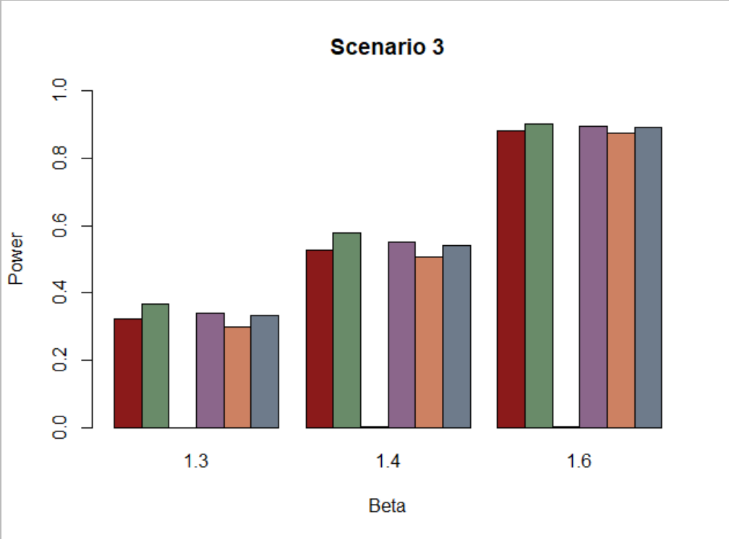

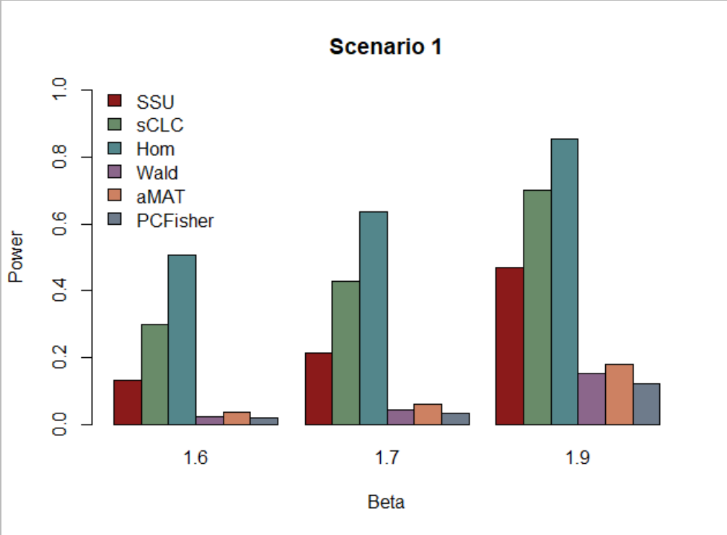

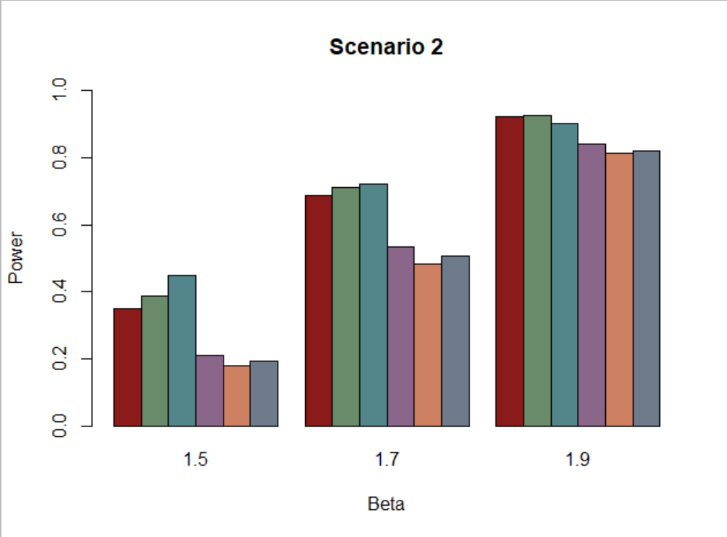


**Figure S2** Power comparisons of the six methods, SSU, sCLC, Hom, Wald, aMAT, and PCFisher for the phenotypic correlation structure of the 70 phenotypes for $\delta={10}^{-4}$ at a significant level of $5\times{10}^{-8}.$


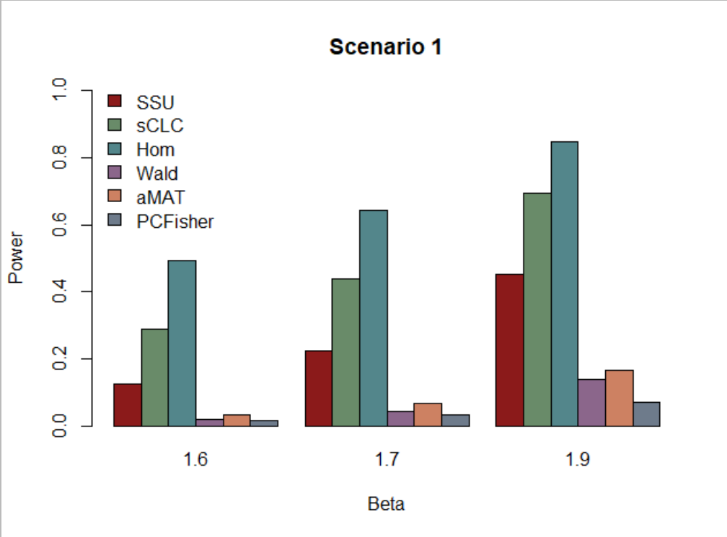

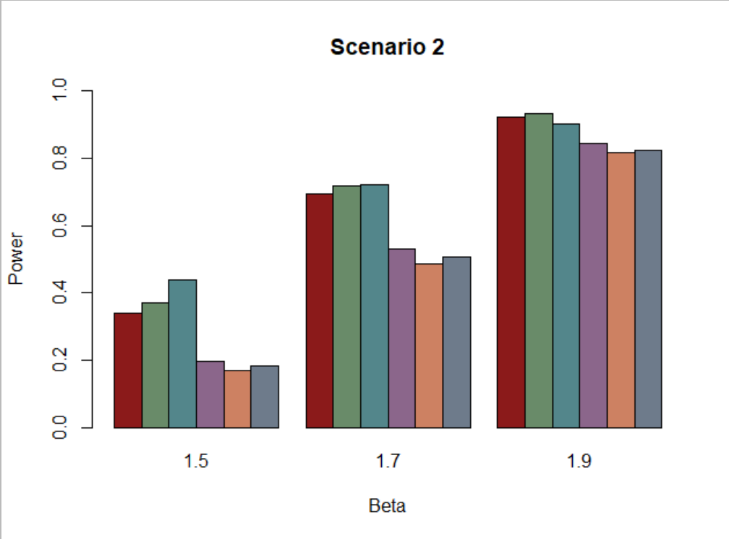

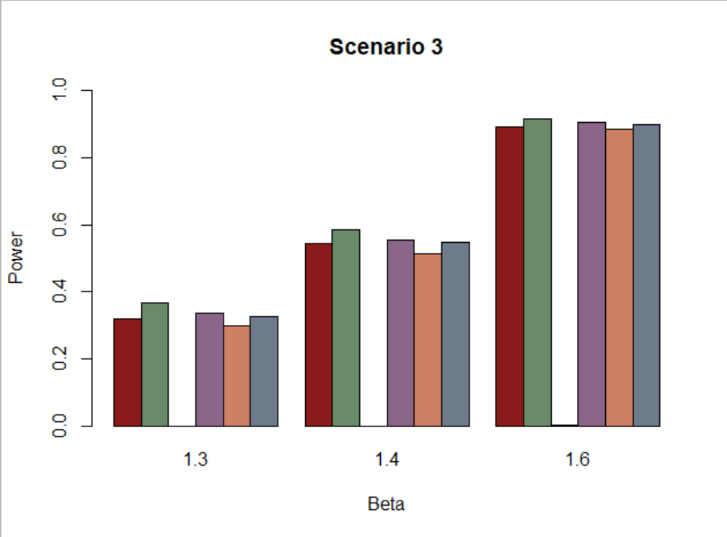

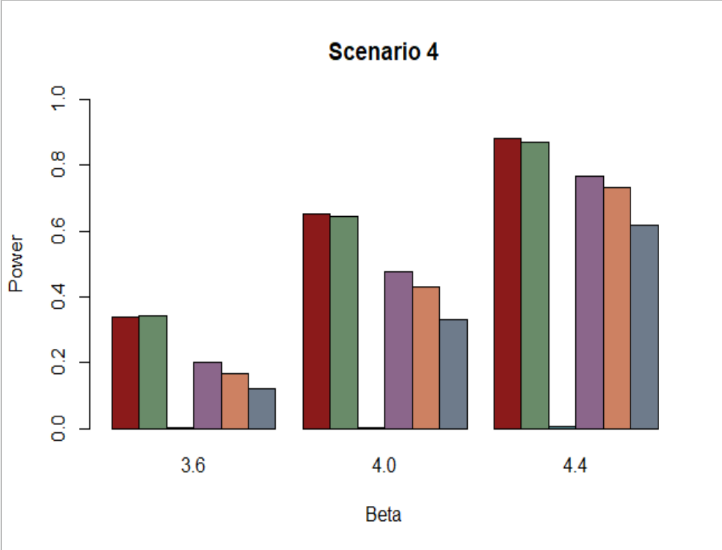


**Figure S3** Power comparisons of the six methods, SSU, sCLC, Hom, Wald, aMAT, and PCFisher for the phenotypic correlation structure of the 40 phenotypes at a significant level of $5\times{10}^{-8}.$


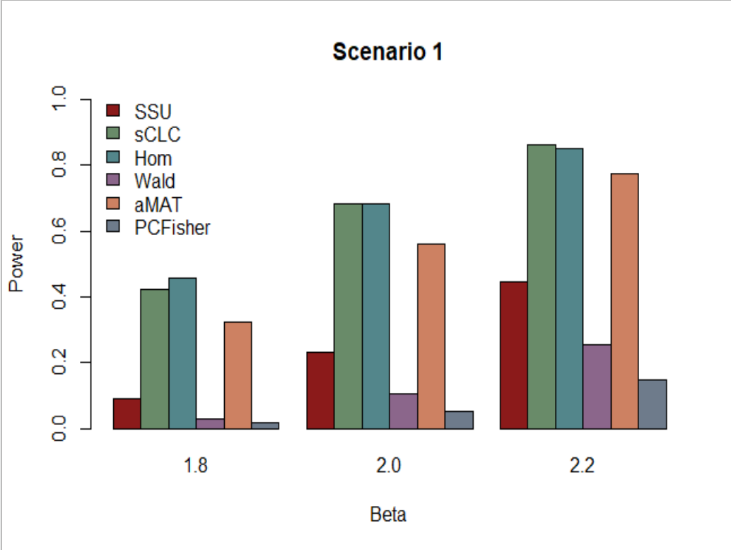

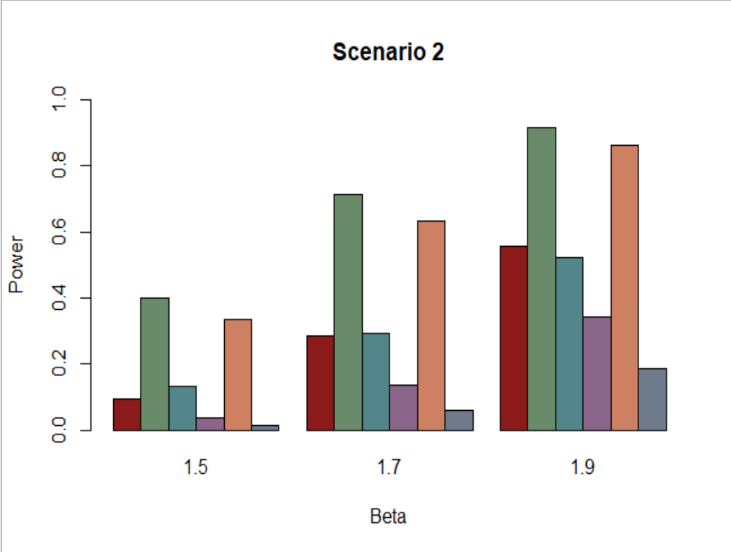

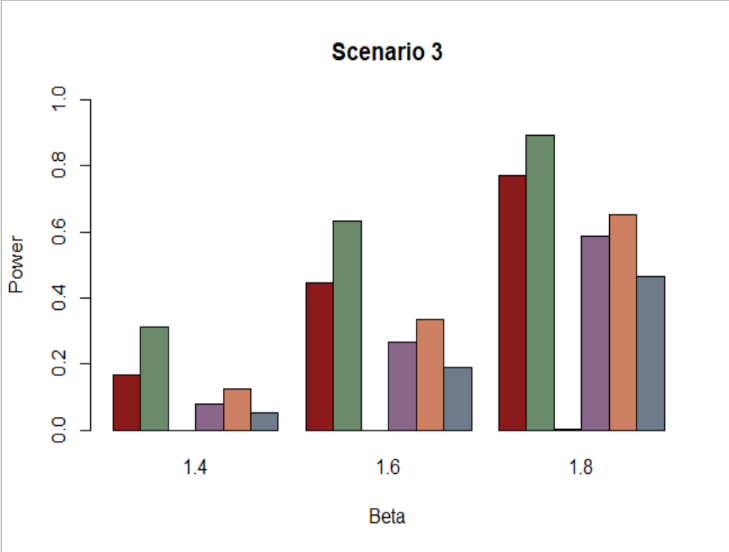

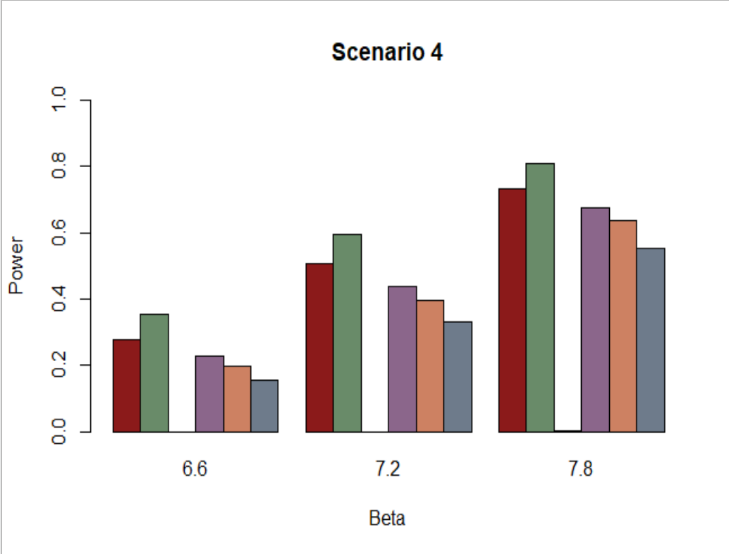

Supplement: Supplementary file 1 — Supplementary Information. [file 41598_2023_30415_MOESM1_ESM.docx]
